# Vaginal cuff dehiscence following controlled ovarian stimulation recognized during egg retrieval

**DOI:** 10.1186/s40738-019-0064-x

**Published:** 2019-11-12

**Authors:** Alexandra Peyser, Avner Hershlag, Antoinette Sakaris, Tomer Singer

**Affiliations:** Division of Reproductive Endocrinology, Department of Obstetrics and Gynecology, North Shore University Hospital, Northwell Health, Zucker School of Medicine at Hofstra/Northwell, 300 Community Drive, Manhasset, NY 11030 USA

**Keywords:** Vaginal cuff dehiscence, Controlled ovarian stimulation, Egg retrieval

## Abstract

**Background:**

Vaginal cuff dehiscence is a rare complication of hysterectomy. Those who choose to undergo controlled ovarian stimulation (COS) and oocyte cryopreservation following hysterectomy must be aware that elevated abdominal pressure from stimulation as well as transvaginal ultrasound use during monitoring may increase the risk of cuff dehiscence.

**Case:**

We present a case of a 25-year-old patient who had undergone a hysterectomy four months prior for endometrial cancer who was found to have vaginal cuff dehiscence which was recognized at the time of egg retrieval after COS. Prompt recognition and appropriate management led to successful treatment.

**Conclusion:**

Patients presenting for oocyte cryopreservation following hysterectomy are at risk for cuff dehiscence. Providers should allow ample time for proper cuff healing prior to COS and oocyte cryopreservation.

## Background

Vaginal cuff dehiscence, the separation of the vaginal incision closed at the initial time of hysterectomy, is one of the most serious complications following hysterectomy. Its incidence ranges between .14 and 4.1% [1], occurring more frequently (1.7%) in hysterectomies for malignant indications and minimal invasive surgeries [1]. Studies have suggested a higher rate of dehiscence in patients who have undergone a robot-assisted approach [2]. Dehiscence of the vaginal cuff after hysterectomy occurs predominantly after coitus in premenopausal women and can be associated with eviscerations of the bowel, adnexa, and omentum [1]. If dehiscence with bowel evisceration is suspected, management consists of immediate imaging and if confirmed, surgical assessment of the overall integrity of the entire bowel. If ischemia is present, bowel resection is almost always necessary.

Pre-menopausal patients who have undergone a hysterectomy with ovarian conservation and desire a genetically related child, may undergo in-vitro fertilization (IVF) with controlled ovarian stimulation (COS) and cryopreservation of oocytes or embryos with the use of a gestational carrier. Studies have demonstrated an earlier decline of ovarian function in pre-menopausal women after hysterectomy due to decreased blood supply to the ovaries following ligation of both utero-ovarian vessels [3]. However, no studies have addressed the optimal time interval necessary between hysterectomy and egg retrieval. Enlarging ovaries during COS, as well as the manipulation during transvaginal egg retrieval, may disrupt the often fragile integrity of the vaginal cuff in women with a recent hysterectomy. Here, we report a case of vaginal cuff dehiscence recognized at the time of egg retrieval four months post robotic hysterectomy for endometrial cancer.

## Case presentation

A 25 year old nulliparous female with a history of stage II endometrial cancer (mixed serous and endometroid type) who underwent a robotic-assisted radical hysterectomy, bilateral salpingectomy and pelvic and para aortic lymph node dissection four months prior, presented to our fertility center desirous of a genetically related child. The patient’s medical history included: asthma (not requiring steroids), bronchitis, juvenile rheumatoid arthritis, and a pituitary adenoma. She was also a carrier of a BRCA1 gene mutation. The patient declined adjuvant chemotherapy in lew of extensive counseling.

On physical exam, the patient was a healthy appearing woman. She had a BMI of 33 and an AMH of 2.7 ng/ml.

The patient received low dose gonadotropins: Human Menopausal Gonadotropin (Menopur®, Ferring Pharmaceuticals, Parsippany, NJ, USA) and FSH (Gonal F®, EMD Serono, Rockland, MA, USA) for 12 days; Cetrorelix acetate (Ganirelex®, GnRH antagonist, EMD Serono, Rockland, MA, USA) was given for the last 6 days. Final oocyte maturation was triggered with choriogonadotropin alfa injection (Ovidrel®, MD Serono, Rockland, MA, USA). The stimulation protocol included Letrozole (Femara®, Novartis, Basel, Switzerland), 2.5 mg twice daily to reduce estrogen levels. Transvaginal and transabdominal ultrasounds were performed daily during COS. On the day of retrieval there were 7 mature follicles on the right ovary ranging from 16 to 25 mm in size and 6 mature follicles on the left ovary ranging from 14 to 19 mm.

**Fig. 1:**
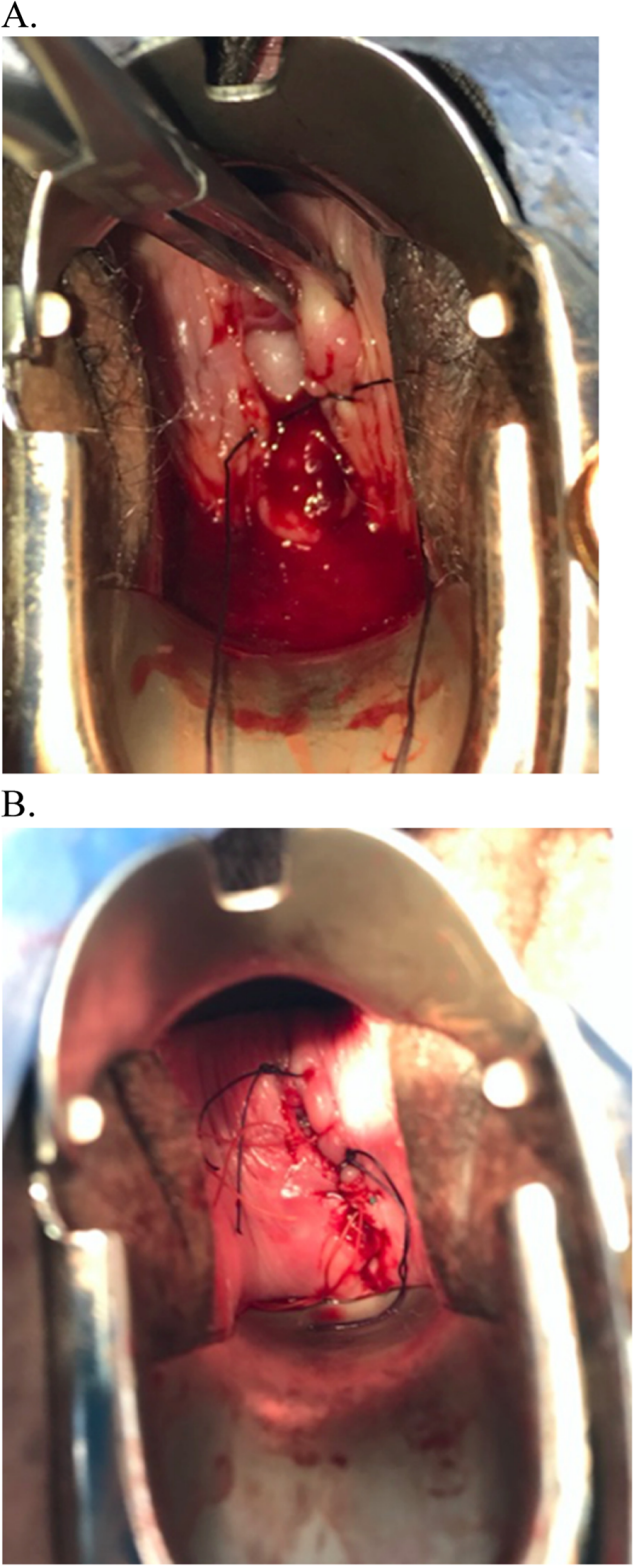
**a**. Vaginal Cuff Dehiscence at the time of egg retrieval. **b**. Vicryl sutures placed at the level of the cuff

During transvaginal oocyte retrieval, bowel was visualized cephalad through a 3 cm separation of the vaginal cuff (Fig. 1). The patient was immediately placed in the Trendelenburg position and the cuff was closed with four interrupted vicryl sutures. Vaginal packing was placed and the patient was sent to the emergency room (ER) for further assessment. CAT scan revealed a small amount of fluid in the pelvis containing high density material. No free air or other evidence of perforation was noted. Repeat physical exam performed by a gynecologic oncologist revealed the vaginal epithelium at the cuff closure to be well perfused; the vicryl sutures were intact and no palpable or visible defect was noted in the vaginal apex. Small pooling at the posterior fornix of clear peritoneal fluid was observed. The white blood cell (WBC) count in the ER was 11.9 and vital signs were stable.

The patient was admitted to the hospital for observation and placed on strict bed-rest for twenty-four hours on antibiotics (Doxycycline, Levofloxacin and Metronidazole). Repeat CAT scan was performed twenty-four hours post retrieval and revealed a small amount of fluid within the pelvis containing high density material. No free air or other evidence of perforation was noted. On post retrieval day one, the patient remained stable. Repeat WBC count was 11.3. The patient was discharged forty-eight hours post retrieval and remained on antibiotics and Letrozole for an additional seven days. One week following egg retrieval, the vaginal cuff remained intact. The patient continued a close follow up with her gynecologic oncologist.

## Discussion and conclusion

Potential risk factors for cuff dehiscence following hysterectomy have been extensively studied. Most studies find minimally invasive surgeries, in particular robotic assisted laparoscopic hysterectomies, to be associated with a higher chance of dehiscence [1, 2]. One study found a 3-fold increase in dehiscence when the indication was a malignancy [3]. In addition, surgeon experience, in particular the years of practice and surgical volume, is inversely proportional to the incidence of cuff separation [3]. Colpotomy, mode of suture, type of suture, and suturing technique have not been associated with an increased incidence [4].

Other non-surgical risk factors associated with vaginal cuff dehiscence include post-operative infection, postmenopausal status, exposure to pelvic radiation, corticosteroid use, penetrative vaginal trauma, previous history of vaginal surgery, and coitus prior to full healing of the cuff [4].

Risk factors may differ between pre and post-menopausal women. In a premenopausal woman, vaginal trauma during coitus, especially the first postoperative event, has been noted as an inciting factor of vaginal cuff dehiscence. The literature describes dehiscence to occur typically 6 weeks to 4 months post hysterectomy [5]. However, sporadic cases have been reported 3 years and even up to 11 years after surgery [6]. In postmenopausal women, chronic pelvic organ prolapse and an increase in abdominal pressure play a major role [4]. Radical hysterectomy is associated with a nine-fold increase in vaginal cuff complications compared with simple hysterectomy [4]. In robotic hysterectomies of gynecologic malignancies, a normal BMI, postoperative chemotherapy, brachytherapy, and early resumption of sexual activities are all risk factors [4]. Obese women (BMI ≥ 30) are significantly less likely to experience dehiscence than women with a BMI < 25 [7].

In our case, the patient underwent a radical robotic hysterectomy for endometrial cancer with vaginal manipulation and had a connective tissue disorder which all could have played a role in the dehiscence. In addition, the increase in abdominal pressure caused by enlarged stimulated ovaries added an additional risk.

The dehiscence was only recognized immediately after the egg retrieval upon cleaning the vaginal wall. However, it is possible to assume that the dehiscence was present prior to initiating any fertility treatment. If the latter were true, the COS in combination with transvaginal ultrasound manipulation exacerbated the already in place dehiscence.

Management of a dehiscence requires immediate evaluation. A bimanuel and speculum exam should be performed. If no evisceration of bowel contents are noted, the patient should be placed in Trendelenberg position and vaginal packing should be placed simultaneously with a Foley catheter. If bowel contents are noted, an attempt to replace it intraperitoneally using sponge sticks should be performed followed by vaginal packing. Abdominal radiographs should be done if possible to rule out the presence of bowel ischemia. A consultation with a general surgeon or a gynecologic oncologist is strongly recommended and surgical repair of the cuff is warrented [4]. Regardless of when the dehiscence occurred in this patient the management would have remained the same.

In patients with a gynecologic cancer requiring hysterectomy and oophorectomy who would like to preserve their fertility, COS may be done preoperatively with egg retrieval prior to surgery with clearance by their oncologist [8]. Another option for these patients is to undergo COS and perform cancer surgery timed concurrently with egg retrieval. Case reports have been published describing this technique with successful outcomes [9, 10]. Although this may pose medical as well as logistical issues, it may be the only chance these patients have for a genetic child in the future.

This patient was a BRCA1 carrier who are not only at risk for breast and ovarian cancer, but have now been shown to be associated with ovarian dysfuction with diminished ovarian reserve [11]. BRCA1 and BRCA2 are part of the family of ataxia-telangiectasis-mutated (ATM) mediated DNA double strand repair genes, and have critical roles in the DNA repair pathway. Inefficient repair causes DNA damage accumulation and contributes to oocyte apoptosis and depletion [12]. BRCA1 patients are recommened to undergo a prophylactic bilateral salpingo-oophorectomy between the ages of 35–40 [13]. These patients face complex challenges and this could have contributed to this patients short interval from surgery to retrieval.

Reproductive Endocrinologists should use individualized management with careful evaluation of the vaginal cuff before and during ovulation induction for medical egg or embryo freezing, with minimal pressure applied to the incision sites during ultrasound monitoring. Assessment of the vaginal cuff pre and post egg retrieval is mandatory. In addition, given the risk of dehischence, it is reasonable to perform a trans-abdominal ultrasound rather than transvaginal during daily monitoring. Vaginal rupture has been reported after transvaginal ultrasonography and vaginal dilator use [2]. An abdominal egg retrieval may also be warrented to preserve the integrity of the cuff if anatomically feasible.

The ideal time between total hysterectomy and egg retrieval for fertility preservation has yet to be established. In patients who present following a diagnosis of a gynecological malignancy (cervical, endometrial and ovarian), there is usually an urgent indication to preserve fertility due to the frequent need for adjuvant chemotherapy or radiation, or in other cases, the need for hysterectomy and bilateral oophorectomy. Therefore waiting, in many cases, is not an appropriate option. Fertility preservation options may not meet the standard of care in certain patient populations with advanced disease and these patients unfortunately must weigh the risks and benefits of their options. Guidelines developed recommend that fertility-preserving approaches are chosen according to the age of the patient, type of cancer and treatment required, the presence or not of a male partner or patient preference for using donor sperm, the time available for fertility preservation intervention and the probability of ovarian metastasis [14]. Fertility sparing surgery may be an option for early-stage cervical cancer with loop excision techniques and radical trachelectomy, allowing preservation of the ovaries and uterus. It is also possibile in stage I epitheilial ovarian cancer, germ cell ovarian tumors and borderline cancers. Hormonal therapy with progesterone is effective in early endometrial cancers [8].

Those who have had a hysterectomy for other indications (i.e endometriosis, leiomyomas, postpartum hemorrhage, pelvic inflammatory disease, transgender reassignment) may opt to delay fertility preservation. However, studies have demonstrated a twofold increased risk for ovarian failure among women undergoing hysterectomy without bilateral salpingo-oophorectomy compared to woman with intact uteri [15]. We suggest an interval of at least 4–6 months between hysterectomy and egg retrieval in benign disease to decrease the risk of dehiscence. If malignancy is present, risks and benefits must be carefully discussed. This is often a difficult decision and must be individualized for each patient.

## Data Availability

Not applicable.

## Abbreviations

COS: Controlled ovarian stimulation
WBC: White Blood Count
